# Maternal autoimmune disease and risk of offspring autism spectrum disorder – a nationwide population-based cohort study

**DOI:** 10.3389/fpsyt.2023.1254453

**Published:** 2023-11-03

**Authors:** Ching-Chu Chen, Ching-Heng Lin, Ming-Chih Lin

**Affiliations:** ^1^Children’s Medical Center, Taichung Veterans General Hospital, Taichung, Taiwan; ^2^Department of Medical Research, Taichung Veterans General Hospital, Taichung, Taiwan; ^3^Department of Post-Baccalaureate Medicine, College of Medicine, National Chung Hsing University, Taichung, Taiwan; ^4^School of Medicine, National Yang Ming Chiao Tung University, Hsinchu, Taiwan; ^5^Department of Food and Nutrition, Providence University, Taichung, Taiwan; ^6^School of Medicine, Chung Shan Medical University, Taichung, Taiwan

**Keywords:** autoimmune disease, Sjogren’s syndrome, rheumatoid arthritis, autism spectrum disorder, prenatal exposure

## Abstract

**Introduction:**

Autism spectrum disorder (ASD) is a group of neurodevelopmental disorders which cause long term social and behavior impairment, and its prevalence is on the rise. Studies about the association between maternal autoimmune diseases and offspring ASD have controversial results. The aim of this study was to investigate whether maternal autoimmune diseases increase the risk of ASD in offspring from a population-based perspective.

**Methods:**

The data sources were Taiwan’s National Health Insurance Research Database (NHIRD) and Taiwan’s Maternal and Child Health Database (MCHD), which were integrated and used to identify newborns whose mothers were diagnosed with autoimmune disease. Newborns were matched by maternal age, neonatal gender, and date of birth with controls whose mothers were without autoimmune disease using a ratio of 1:4 between 2004 and 2019. Data on diagnoses of autoimmune disease and autism spectrum disorders were retrieved from NHIRD. Patients who had at least 3 outpatient visits or at least 1 admission with a diagnosis of autoimmune disease and autism spectrum disorders were defined as incidence cases. The risks of ASD in offspring were compared between mothers with or without autoimmune disorders.

**Results:**

We identified 20,865 newborns whose mothers had been diagnosed with autoimmune disease before pregnancy and matched them at a ratio of 1:4 with a total of 83,460 newborn whose mothers were without autoimmune disease, by maternal age, neonatal gender, and date of birth. They were randomly selected as the control group. The cumulative incidence rates of autism spectrum disorders (ASD) were significantly higher among the offspring of mothers with autoimmune diseases. After adjusting for cofactors, the risk of ASD remained significantly higher in children whose mother had autoimmune diseases. Regarding to specific maternal autoimmune disease, Sjögren’s syndrome and rheumatoid arthritis were both associated with elevated risks of ASD in offspring.

**Conclusion:**

Mother with autoimmune disease might be associated with increasing the risk of autism spectrum disorder in offspring.

## Introduction

Autism spectrum disorder (ASD) is a set of neurodevelopmental disorders which cause persistent impairments in social interaction, social communication, as well as restrictive and repetitive patterns of behavior, interests, and activities (1). The population prevalence of ASD is on the rise in both Western and Eastern nations (2–6). It is estimated that approximately one in every hundred children are diagnosed with ASD and the prevalence rate is four times greater in males than in females (7). The exact pathogenesis and mechanism of ASD is incompletely understood, but several factors have been proposed in the pathogenesis of autism spectrum disorders, including genetic factors (8, 9), neurobiological factors (10, 11), parental factors (12), environmental and perinatal factors (13, 14), maternal medication use during pregnancy (14), and maternal autoimmune disease (15, 16). The core presentations of autism spectrum disorders are not only impaired social communication and interaction but also restricted and repetitive behavior, interest, and activities (17). The majority of individuals with ASD need continuous educational support (18). ASD has been reported to be associated with significantly increased risk of mortality (19).

Decades of research into the pathomechanism contributing to the development of ASD have revealed that maternal immune activation may play a crucial role. This activation can be triggered by both acute and chronic systemic inflammation (15). Recent evidence suggests that certain factors associated with systemic chronic inflammation, such as obesity, gut microbiome alterations, gestational diabetes (GDM), pre-eclampsia, smoking, exposure to pollution, low socioeconomic status, depression, stress, autoimmune diseases, asthma, and infection, contribute to maternal immune activation. Some of these chronic inflammatory conditions during pregnancy are significantly associated with an elevated risk of neurodevelopmental disorders in offspring, including ASD, attention deficit hyperactivity disorder, and Tourette syndrome (16, 20).

Previous epidemiology studies have shown maternal conditions during pregnancy might increase the incidence of certain diseases in offspring (21–23). However, research on the relationship between maternal autoimmune diseases and ASD in offspring have yielded conflicting results. Some studies have found an increased risk of ASD in children born to mothers with autoimmune diseases (24, 25). However, other studies found no association (26, 27). Therefore, the aim of this study was to investigate whether maternal autoimmune diseases increase the risk of ASD in offspring from a population-based perspective.

## Materials and methods

### Study design and data source

This was a nationwide, population-based, cohort study. Data were mainly derived from Taiwan’s Maternal and Child Health Database (MCHD), which is maintained by the Health and Welfare Data Science Center (HWDC) of the Ministry of Health and Welfare (MOHW). The MCHD is linked to the Taiwan Birth Registration Database (BRD), Birth Certificate Application (BCA), National Register of Death (NRD), and the National Health Insurance Research Database (NHIRD), providing integrated medical claims data of offspring and their parents. The National Health Insurance (NHI) program, a government-run, single-payer regime with mandatory enrollment, was launched in Taiwan on March 1, 1995. As of December 31, 2021, 99.99% of Taiwan’s population of nearly 23.5 million were enrolled under this program. The NHIRD was established in 2002 based upon all-inclusive claims data from the NHI (28, 29). It contains each patient’s residence, registration, demographic characteristics, examinations, diagnoses, procedures, surgeries, medication prescriptions, medical expenditure, inpatient services, and outpatient services. The main data sources from the NHIRD used in our analysis were the inpatient expenditures by admission (DD) and the ambulatory care expenditures by visit (CD) files. We integrated these databases to establish detailed linked profiles between mothers and children from the TMCHD. All diagnoses in the NHIRD are coded using the International Classification of Diseases, Ninth Revision, Clinical Modification (ICD-9-CM) before September 20, 2015, and the International Classification of Diseases, Tenth Revision, Clinical Modification (ICD-10-CM) format after October 01, 2015. To protect data privacy, investigators are requested to conduct on-site analysis at an HWDC through remote connection to MOHW servers. This study protocol was approved by the institutional review board of Taichung Veterans General Hospital (CE17178A-5). Since all patient data had been anonymized before analysis, the review board waived the need for written informed consent.

### Case identification and study designs

The cohort included births of primipara mothers between 2004 and 2019 (only the first child during the study period in a family was included). Births with clear information on current place of residence was included, and patients who had a congenital anomaly (ICD-9-CM code 759.9/ ICD-10-CM code Q89.9) were excluded. Children in this study were recruited at various points between 2004 and 2019 and followed until 2020. They were followed for a duration of 1 to 15 years based on when they were initially recruited. These children were further divided into two groups: mothers with autoimmune disease and mothers without autoimmune disease. Autoimmune diseases were identified by ICD-9-CM codes and ICD-10-CM codes in outpatient medical records, with at least 3 visits, or inpatient medical records, with at least 1 visit. These autoimmune diseases were as follows: systemic lupus erythematosus (ICD-9-CM code 710.0/ ICD-10-CM code M32.10, M32.19, M32.8, M32.9), systemic sclerosis (ICD-9-CM code 710.1/ ICD-10-CM code M34.0, M34.1, M34.89, M34.9), Sjogren’s syndrome (ICD-9-CM code 710.2/ ICD-10-CM code M35.00, M35.01, M35.09), dermatomyositis (ICD-9-CM code 710.3/ ICD-10-CM code M33.00, M33.09, M33.10, M33.19, M33.90, M33.91, M33.99), polymyositis (ICD-9-CM code 710.4/ ICD-10-CM code M33.20, M33.29), rheumatoid arthritis (ICD-9-CM code 714.0/ ICD-10-CM code M05.70, M06.9, M08.00). Retrieval of ASD diagnosis was done according to ICD-9-CM codes and ICD-10-CM codes in outpatient medical records, with at least 3 visits, or inpatient medical records, with at least 1 visit. The codes included ICD-9-CM codes 299.00, 299.01, 315.9, and ICD-10-CM codes F84.0, F84.8, F84.9. In addition, we also collected the NHI premiums as a proxy for the family’s socioeconomic circumstances, because premiums are calculated based on monthly income.

### Statistics

All quantitative data were expressed as either frequency or percentage. Categorical variables were compared using the chi-square test. We employed survival analysis, including Kaplan–Meier estimate and Cox regression model to overcome the problem of varying follow up periods. To control potential confounding factors, multiple Cox regression model was adapted. Maternal age, family income, urbanization, maternal comorbidity, pregnancy-related complication, mode of delivery, neonatal gender, month of birth date, number of babies, birth weight, and gestational age were adjusted by Cox regression model. All data were analyzed using the SAS statistical package (version 9.4; SAS Institute, Cary, NC, United States). A *p* value less than 0.05 was considered statistically significant.

## Results

### Baseline characteristics of offspring of mothers with autoimmune disease and age-matched controls

**Figure 1:**
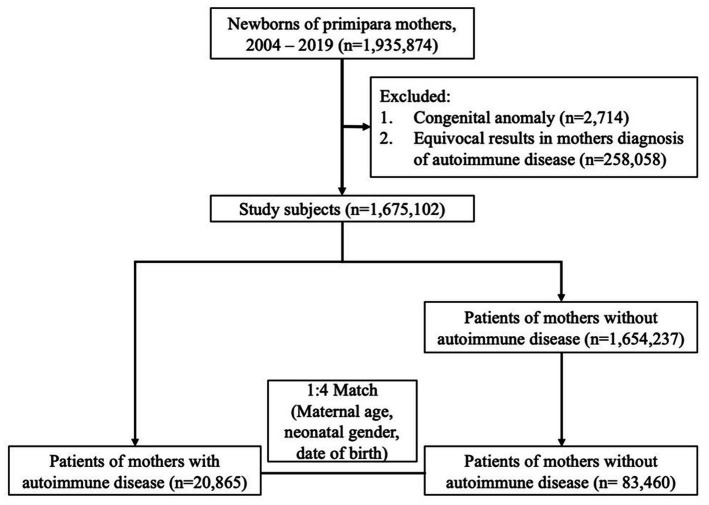
Composition of the study cohort.

In total, 1,675,102 singleton newborns born in Taiwan from 2004 to 2019 were identified and followed up until December 2020. Among the mothers of these children, 20,865 had autoimmune disease and were categorized in the autoimmune disease group. A total of 83,460 mothers without autoimmune disease, matched at a ratio of 1:4 by maternal age, neonatal gender, and date of birth, were randomly selected to serve as the control group (Figure 1). Children in the maternal autoimmune disease group and those in the control group had similar distributions of maternal age, urbanization, maternal pregnancy-related complications, including gestational hypertension, gestational diabetes mellitus, as well as neonatal gender, and month of birth date. Family income was higher in the maternal autoimmune disease group. Mothers with autoimmune disease had a greater incidence of allergic comorbidity (asthma, allergic rhinitis, atopic dermatitis), pregnancy-related complications (pre-eclampsia or eclampsia, placenta previa or abruptio placentae, anemia) and they tended to be more likely to deliver a preterm baby than mothers without autoimmune disease (Table 1).

**Table 1:** Baseline characteristics of the offspring of mothers with autoimmune disease and mothers without autoimmune disease.

Characteristics	Mothers with autoimmune disease (*n* = 20,865) (%)		Mothers without autoimmune disease (*n* = 83,460) (%)		Total	*p*-value
**Maternal age**						1.00
<25	1,108	(5.3)	4,432	(5.3)	5,540	
25–29	5,251	(25.2)	21,004	(25.2)	26,255	
30–34	8,875	(42.5)	35,500	(42.5)	44,375	
≥35	5,631	(27.0)	22,524	(27.0)	28,155	
**Family income**						<0.001
$ ≤ 18,780	3,605	(17.3)	16,796	(20.1)	20,401	
$18781–27,600	7,227	(34.6)	30,200	(36.2)	37,427	
$27601–42,000	5,344	(25.6)	20,115	(24.1)	25,459	
$ > 42,000	4,689	(22.5)	16,349	(19.6)	21,038	
**Urbanization**						0.15
Urban	13,605	(65.2)	54,029	(64.7)	67,634	
Suburban	2,428	(11.6)	9,579	(11.5)	12,007	
Rural	4,832	(23.2)	19,852	(23.8)	24,684	
**Maternal comorbidities**						
Asthma	1,518	(7.3)	3,848	(4.6)	5,366	<0.001
Allergic rhinitis	9,209	(44.1)	25,816	(30.9)	35,025	<0.001
Atopic dermatitis	1,673	(8.0)	3,576	(4.3)	5,249	<0.001
**Pregnancy-related complications**						
Gestational hypertension	56	(0.3)	180	(0.2)	236	0.15
Gestational diabetes mellitus	418	(2.0)	1,580	(1.9)	1,998	0.30
Pre-eclampsia or eclampsia	145	(0.7)	452	(0.5)	597	0.009
Placenta previa or abruptio placentae	427	(2.0)	1,290	(1.5)	1,717	<0.001
Anemia	211	(1.0)	592	(0.7)	803	<0.001
**Mode of delivery**						<0.001
Vaginal delivery	12,449	(59.7)	51,721	(62.0)	64,170	
Cesarean section	8,416	(40.3)	31,739	(38.0)	40,155	
**Neonatal Gender**						1.00
Female	10,099	(48.4)	40,396	(48.4)	50,495	
Male	10,766	(51.6)	43,064	(51.6)	53,830	
**Month of birth date**						1.00
1–3	4,933	(23.6)	19,732	(23.6)	24,665	
4–6	4,978	(23.9)	19,912	(23.9)	24,890	
7–9	5,335	(25.6)	21,340	(25.6)	26,675	
10–12	5,619	(26.9)	22,476	(26.9)	28,095	
**Number of babies**						<0.001
Singleton	20,228	(96.9)	81,449	(97.6)	101,677	
Multiple	637	(3.1)	2,011	(2.4)	2,648	
**Birth weight**						<0.001
≥2,500	17,960	(86.1)	76,267	(91.4)	94,227	
2000–2,499	2,012	(9.6)	5,414	(6.5)	7,426	
<2000	893	(4.3)	1,779	(2.1)	2,672	
**Gestational age**						<0.001
≥37wks	18,119	(86.8)	76,273	(91.4)	94,392	
32–36 + 6wks	2,400	(11.5)	6,491	(7.8)	8,891	
26–31 + 6wks	305	(1.5)	578	(0.7)	883	
<26wks	41	(0.2)	118	(0.1)	159	

### Cumulative incidence rates of autism spectrum disorders

**Figure 2:**
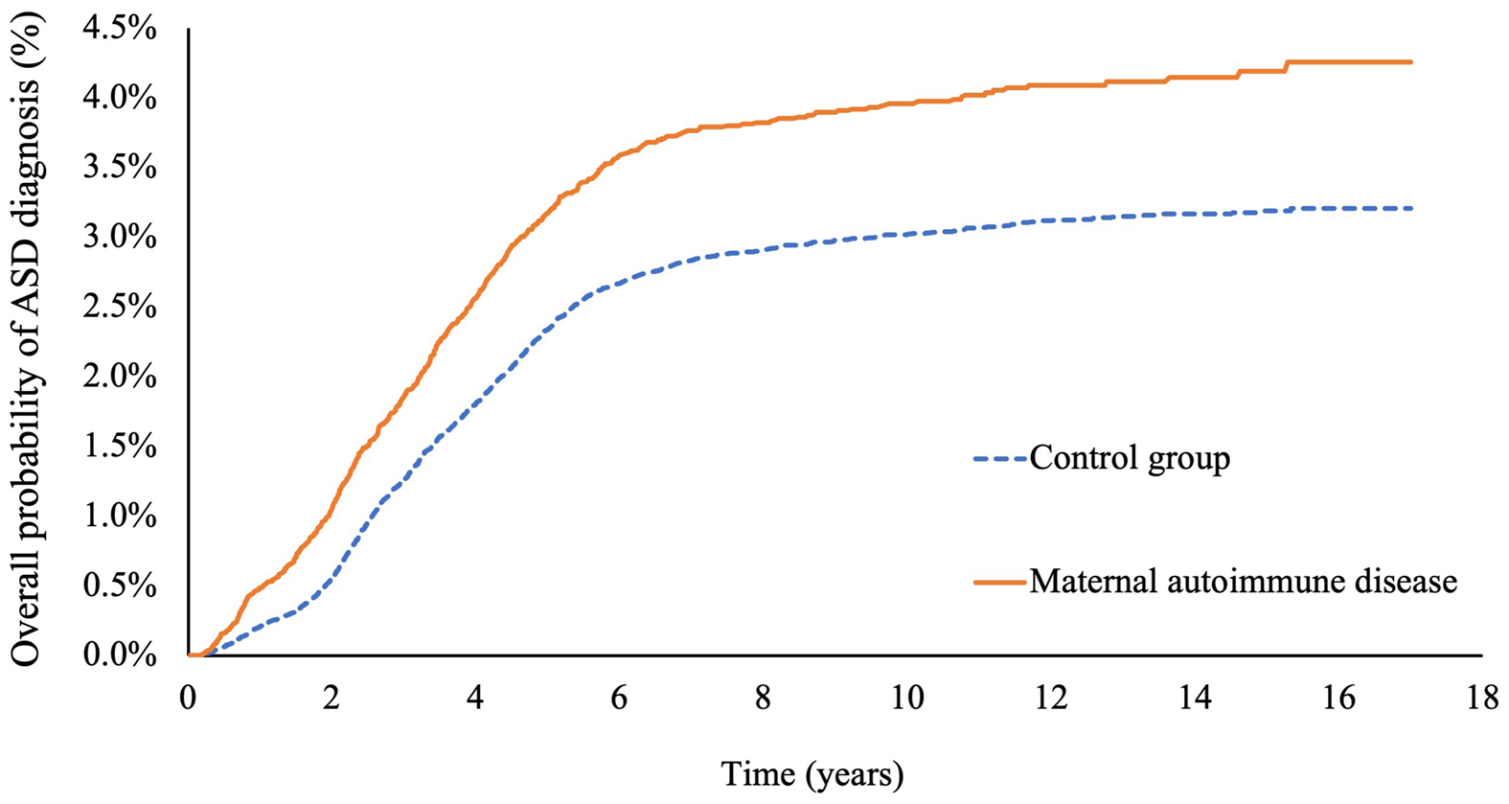
Cumulative risks of autism spectrum disease in offspring with or without maternal autoimmune disease.

The cumulative incidence rates of autism spectrum disorders (ASD) are illustrated in Figure 2. The unadjusted cumulative incidence rates were statistically significantly higher for the offspring of mothers with autoimmune diseases in comparison with the control groups (incidence rate: 4.26% vs. 3.20%, *p* value <0.001).

### Risk of autism spectrum disorders in offspring after adjusting for cofactors by Cox regression model

After adjusting for potential confounding factors by Cox regression model, the hazard ratio for ASD in the offspring of mothers with autoimmune disease was 1.25 (95% CI: 1.15–1.37). Among the cofactors, maternal age greater or equal to 35-year-old, living in urban area, low family income, maternal allergic rhinitis, and gestational diabetes mellitus were associated with higher risks of ASD in offspring. Autism spectrum disorders were more common in male babies. Births between April and June, low birth weight, and premature delivery were also correlated with a higher incidence of ASD (Table 2).

**Table 2:** Multivariable analysis of factors associated with ASD in offspring.

Variables	HR	95%CI		*p*-value
**Mothers with autoimmune diseases**	1.25	1.15	1.37	<0.001
**Maternal age**				
<25	1.00			
25–29	0.87	0.73	1.05	0.14
30–34	1.08	0.91	1.29	0.39
≥35	1.23	1.03	1.47	0.025
**Family income**				
$ ≤ 18,780	1.00			
$18781–27,600	0.79	0.71	0.87	<0.001
$27601–42,000	0.74	0.66	0.82	<0.001
$ > 42,000	0.74	0.65	0.83	<0.001
**Urbanization**				
Urban	1.00			
Suburban	0.84	0.74	0.96	0.007
Rural	0.91	0.83	1.00	0.05
**Maternal comorbidities**				
Asthma	1.16	0.99	1.36	0.07
Allergic rhinitis	1.11	1.02	1.20	0.011
Atopic dermatitis	0.96	0.81	1.15	0.66
**Pregnancy-related complications**				
Gestational hypertension	0.78	0.33	1.89	0.59
Gestational diabetes mellitus	1.32	1.03	1.68	0.027
Pre-eclampsia or eclampsia	0.83	0.52	1.33	0.44
Placenta previa or abruptio placentae	0.98	0.76	1.26	0.87
Anemia	1.24	0.86	1.77	0.25
**Mode of delivery**				
Vaginal delivery	1.00			
Cesarean section	1.06	0.98	1.14	0.17
**Neonatal gender**				
Female	1.00			
Male	2.75	2.52	2.99	<0.001
**Month of birth date**				1.00
1–3	1.00			
4–6	1.14	1.03	1.27	0.014
7–9	1.03	0.93	1.15	0.56
10–12	0.90	0.81	1.00	0.06
**Number of babies**				
Singleton	1.00			
Multiple	1.02	0.84	1.25	0.82
**Birth weight**				
≥2,500	1.00			
2000–2,499	1.40	1.21	1.63	<0.001
<2000	2.46	1.95	3.09	<0.001
**Gestational age**				
≥37wks	1.00			
32–36 + 6wks	1.23	1.07	1.41	0.004
26–31 + 6wks	2.00	1.48	2.69	<0.001
<26wks	5.23	3.12	8.76	<0.001

CI, confidence interval; HR, hazard ratio.

### Specific maternal autoimmune diseases and risk of autism spectrum disorders in offspring

**Figure 3:**
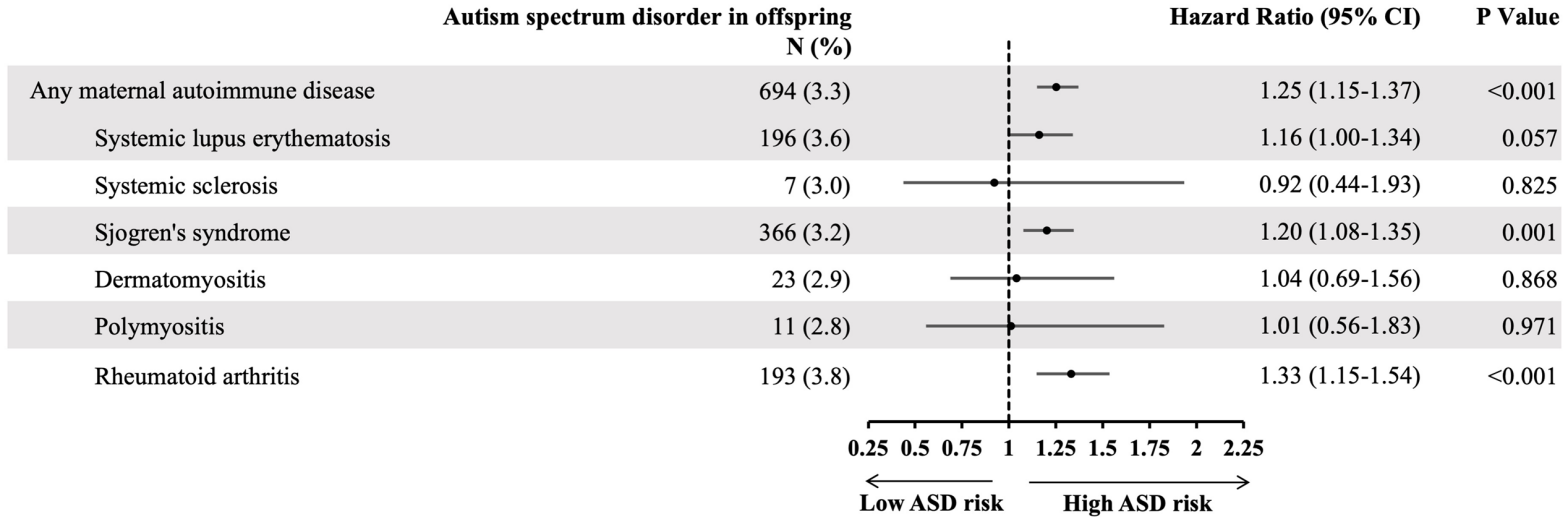
The hazard ratio of specific maternal autoimmune disease and risk of offspring autism spectrum disorder, adjusting cofactors by Cox regression models. Adjusted cofactors including: maternal age, family income, urbanization, maternal comorbidity, pregnancy-related complication, mode of delivery, neonatal gender, month of birth date, number of babies, birth weight, and gestational age.

We further analyzed the ASD risk of each specific maternal autoimmune disease. The results demonstrated that mothers with Sjögren’s syndrome (HR: 1.2, 95% CI: 1.08–1.35) or rheumatoid arthritis (HR: 1.33, 95% CI: 1.15–1.54) specifically tended to have a greater likelihood of giving birth to offspring with ASD (Figure 3).

## Discussion

This nationwide, population-based, longitudinal follow-up study investigated the association between prenatal exposure to maternal autoimmune disease and the risk of autism spectrum disorders in offspring. Our study results revealed children born to mothers with autoimmune disease, especially Sjogren’s syndrome and rheumatoid arthritis, had a higher cumulative risk of ASD.

Chen et al. conducted a meta-analysis which concluded that maternal autoimmune diseases were associated with a 34% increased risk of ASD in offspring (24). However, most of the included research consisted of case–control studies. Recently, a Danish cohort study, which recruited 2,254,234 singleton infants, analyzed the association between a wide range of maternal autoimmune diseases diagnosed before childbirth and the offspring’s full spectrum of mental disorders. It concluded that risk of childhood autism increased after exposure to maternal autoimmune diseases (25). Nevertheless, several cohort studies have different conclusions. In one such investigation, which was a nationwide, population-based, cohort study, Taiwan’s National Health Insurance Research Database (NHIRD) was used as the data source. The authors identified 708,517 family triads (father–mother–child) between 2001 and 2008 and followed them until the end of 2011 to calculate the risk of attention deficit hyperactivity and autism spectrum disorders among the children of parents with autoimmune diseases. The results revealed that pediatric ASD was only associated with paternal inflammatory bowel disease and ankylosing spondylitis (25). In the study, out of a total of 708,517 mothers, only 1990 had any form of autoimmune disease. The number might be underestimated because of differences in exposure definitions and imprecise data links between children and mothers. The statistical power was therefore reduced. Furthermore, the study enrolled children born from 2001 to 2008 and only followed them up for 3 years. Our study incorporated kinship information from Taiwan’s Maternal and Child Health Database (TMCHD) to accurately identify links between mothers and their children. Another study was also a population-based, cohort study that investigated the association between allergic and autoimmune diseases with ASD and ADHD within families. No link was found between maternal autoimmune disease and risk of ASD in their children (26). The study included children born from 2004 to 2016, who were 5 years old or older in 2016 and were followed up until 2017. However, using age of least 5 years old as one of the inclusion criteria might have resulted in an underestimation of the population of autism spectrum disorders, because a nationwide study in Taiwan reported that most of subjects with ASD were diagnosed in the preschool period (30).

Researchers have explored the pathomechanism of ASD over the past few decades in order to discover novel and more effective therapeutic targets. Although the pathogenesis of ASD is not fully understood, the consensus is that the cause of ASD is multifactorial, including genetic factors, environmental factors, parental factors, perinatal factors, and maternal autoimmune disease. Recently, animal models and integrated relevant studies have proposed that genetic factors, environmental factors, innate immune dysfunction and maternal autoimmunity contribute to the pathomechanism of ASD (31, 32). ASD is highly heritable and has been found to be linked to hundreds of diverse genetic polymorphisms. Numerous novel ASD-associated genetics variants have been identified by Genome Wide Association Studies (GWAS) (33, 34). The convergence of many different genetic etiologies may be involved in the disruption of intracellular signaling of MET receptor tyrosine kinases (RTKs), which play a crucial role in the signaling system, as they modulate neuronal growth, facilitate functional maturation, and establish effective brain circuits, especially in the brain regions that help to govern advanced cognitive abilities, social competence, and executive functioning (9, 35). However, the phenomenon of phenotypic heterogeneity with common functional variants in ASD children has been observed, and hence researchers speculated that gene mutations alone might be insufficient to contribute to all symptoms of ASD, though they may increase the risk in genetically susceptible individuals. More recently, the concept of maternal immune activation (MIA) has been proposed. MIA is thought to affect fetal neurodevelopment through inflammatory and epigenetic mechanisms, and ultimately leads to the development of ASD in children. Moreover, MIA is triggered by genetic mutation, environmental factors, maternal acute and systemic chronic inflammation, which includes genetic, smoking, obesity, gestational diabetes, pre-eclampsia, depression, psychosocial stress, pollution, low socioeconomic status, asthma, and autoimmune diseases (20). According to Peng et al. (35), there are several ASD-associated genes that encode components of the immune system. One of the associated genes is the human leukocyte antigen (HLA) genes, which are located on chromosome 6 and have been found involved in immune dysfunction and autoimmune diseases. It also plays a role in neural cell interactions, synaptic function, cerebral hemispheric, specialization, central nervous system (CNS) development and even neurological disorders. Based on a substantial body of evidence suggesting that the immune system contributes to the development of ASD in specific individuals, several studies were subsequently conducted to investigate the relationship between the HLA gene and ASD, revealing a strong genetic association with the disorder. Especially that strong associations were identified in Class I region HLA- A2, class II region DRβ1*04 allele, DRβ1*11-DQβ1*07, class III region C4B null allele (36, 37). Besides, Lee et al. found that boys diagnosed with autism, as well as their mothers, exhibited a notably higher prevalence of HLA-DR4 compared to the normal control group. This suggests that maternal-fetal immune interactions could potentially influence fetal brain development and, consequently, lead to autism (38). The other well-studied immune genes strongly associated with ASD is MET, as mentioned above. The MET gene encodes for hepatocyte growth factor receptors, which promote differentiation and proliferation of hematopoietic cells and exert broad anti-inflammatory effects (35). A reduced expression of the MET gene in the temporal lobe was found in a post-mortem transcriptome analysis of individuals with ASD (39) and MRI imaging revealed reduced structural and functional connections in the temporoparietal lobes of individuals with ASD with the MET variant (40). Animal models and several epidemiological studies have demonstrated maternal factors, such as exposure to pollution, obesity, stress, asthma, and infection, result in behavioral and transcriptional changes in offspring, through cytokine signaling mechanisms mediated by the placenta (41–44).

As for maternal autoimmune disease, MIA in the prenatal and early post-natal periods also plays a crucial role, comprising multiple pathways, in the development of ASD in offspring. It has been suggested that mothers with autoimmune disease harbor much more brain-reactive antibodies and antibodies could be transmitted to the fetus passively through the placenta. Evidence supporting this hypothesis has been found in animal model studies and large cohort studies conducted by Brimberg et al. (45). Theoretically, fetal brain-reactive antibodies or proinflammatory cytokine released while mother was infected during pregnancy would lead to the activation of maternal TH17 cells. These activated TH17 cells would affect placenta function and causes damage. It allows the antibodies and cytokines to pass through to the developing fetus and reach the fetal brain during early gestation as the blood–brain barrier (BBB) is immature until the postnatal period (46). Most importantly, these fetal brain-reactive antibodies would be triggered by specific protein antigens in the fetal brain. This interaction could ultimately affect fetal neurodevelopment because these proteins, such as lactate dehydrogenase A and B (LDH-A and LDH-B), collapsing response mediator proteins 1 and 2 (CRMP1 and CRMP2), and stress-induced phosphoprotein 1 (STIP1), are involved in different aspects of the neuronal function (47–49). Evidence from several experimental animal model studies shows that transplacental passage of fetal brain-reactive antibodies increase the risk of developing ASD. In the studies, pregnant mice and Rhesus monkeys were injected with anti-brain IgG antibodies from the mothers of children with ASD and the behaviors of their offspring were then observed (50–52). Another mechanism of greater risk of ASD in offspring that has been proposed involves increased levels of maternal pro-inflammatory cytokines, induced by viral or bacterial infections (MIA), during the gestational period. Taken together, the findings of several animal model and cohort studies have demonstrated that overexpression of cytokines IL-6, IL-17, and IL-1β in the developing brain could lead to redundant inflammation, dysfunction of microglial activation in the brain, and abnormal cortical development, phenomena that closely resemble the changes found in ASD patients (53, 54).

Changes in the maternal microbiome through MIA leading to activation of IL-17 has recently been suggested as a novel pathomechanism of pediatric ASD (55). Dysregulation of the gut microbiome involving the brain-gut-microbiome axis is also thought to be associated with the development of neurological and psychiatric disorders, including ASD. Multiple factors appear to play a role, such as mode of delivery, probiotics, stress, circadian clock system, occupational/environmental exposure, and diet (56). There did not appear to be any significance difference between two modes of delivery in our multivariable analysis of ASD children, but further investigation is warranted to better understand the relationship between ASD and the microbiome. The possible reasons why family income and urbanization play roles in the incidence of ASD are explained as following. Low-income families might have higher risk of psychosocial stress, depression, and poor lifestyles, etc. These factors might contribute to maternal systemic chronic inflammation which has been proposed to affect fetal neurodevelopment, through inflammatory and epigenetic mechanisms, consequently, result in ASD. Moreover, family living in urban areas might have better medical accessibility and parents might also have more willingness to have their children seek medical advice and get diagnosis of ASD. Overall, our analysis of the published studies over the past few decades indicates that maternal autoimmune disease is associated with an increased risk of ASD in offspring.

Our study had several strengths. First, we used a large, national, population-based sample and the follow-up period spanned a comparatively long duration. Second, we used integrated data from Taiwan’s Maternal and Child Health Database (TMCHD) to more precisely identify the kinship between each of the enrolled children and their mothers. Furthermore, the medical service utilization history according to the National Health Insurance Research Database (NHIRD) was available for all cases in our study, which helped minimize the potential for selection or recall bias. Accordingly, it was possible to adequately investigate our hypothesis. However, there were still some potential limitations in our study. First, some factors (gestational diabetes, pre-eclampsia, low-income status, and asthma) would produce bias in association effects, but if all these factors were included as exclusion criteria, it might limit the external validity. Therefore, to overcome the issue, we adjusted these factors by Cox regression models, as we had a sufficient study population number, to more comprehensive assessment of the correlation between maternal autoimmune disease and the risk of offspring developing ASD. Second, the NHIRD did not include data on certain covariates, such as personal lifestyle (maternal obesity, smoking, status of psychosocial stress, unhealthy diet etc.), education level, laboratory data, genetic sequencing, pollution exposure and environmental factors. Additionally, information regarding the treatment of autoimmune diseases, such as the response to treatment, and the disease status, which could potentially serve as confounding factors, were unavailable by retrieving ICD codes from NHIRD. Third, we did not further analyze if there had any of the neurodevelopmental disorders included in ASD more associate to the mother’s autoimmunity, because we could not differentiate each neurodevelopmental disorders in ASD based on ICD-9 code and ICD-10 code retrieved from Taiwan National Health Insurance Research Database (NHIRD). Fourth, we did not analyze the effect of paternal autoimmune disease, which might be one of the biases. Fifth, there might have been some misclassification bias of maternal autoimmune disease and childhood autism spectrum disorders because diagnoses were mainly retrieved from the health insurance claims data.

## Conclusion

Maternal autoimmune disease was associated with increased risk of ASD in offspring. Further research is required to elucidate the precise pathomechanism of ASD.

## Data availability statement

The datasets presented in this article are not readily available because data release is not allowed by the National Health Insurance Research Database. Requests to access the datasets should be directed to Dr. Ching-Heng Lin/ epid@vghtc.gov.tw.

## Ethics statement

The studies involving humans were approved by the Institutional Review Board of Taichung Veterans General Hospital. The studies were conducted in accordance with the local legislation and institutional requirements. Written informed consent for participation was not required from the participants or the participants’ legal guardians/next of kin in accordance with the national legislation and institutional requirements.

## Author contributions

C-CC: Conceptualization, Investigation, Writing – original draft. C-HL: Data curation, Formal analysis, Writing – review & editing. M-CL: Conceptualization, Funding acquisition, Methodology, Project administration, Supervision, Validation, Writing – review & editing.
